# Novel Ultrastructural Insights into the Clear-Cell Carcinoma of the Pancreas: A Case Report

**DOI:** 10.3390/ijms25084313

**Published:** 2024-04-13

**Authors:** Valentina Giansante, Luca Di Angelo, Chiara Calabrese, Paolo De Sanctis, Paolo Regi, Filippo Maria Martelli, Gianmarco Stati, Rossano Lattanzio, Saverio Alberti, Emanuela Guerra, Roberta Di Pietro

**Affiliations:** 1 Department of Medicine and Aging Sciences, Section of Biomorphology, “G. d’Annunzio” University of Chieti-Pescara, Via dei Vestini, 31, 66100 Chieti, Italy; valentina.giansante@unich.it (V.G.); luca.diangelo@studenti.unich.it (L.D.A.); chiaracalabrs@gmail.com (C.C.); paolo.desanctis@studenti.unich.it (P.D.S.); gianmarco.stati@unich.it (G.S.); emanuela.guerra@unich.it (E.G.); 2 “P. Pederzoli” Hospital, Via Monte Baldo, 24, 37019 Peschiera del Garda, Italy; paoloregichirurgo@gmail.com (P.R.); filippomartelli95@gmail.com (F.M.M.); 3 Department of Innovative Technologies in Medicine & Dentistry, “G. d’Annunzio” University of Chieti-Pescara, Via dei Vestini, 31, 66100 Chieti, Italy; rossano.lattanzio@unich.it; 4 Laboratory of Cancer Pathology, Center for Advanced Studies and Technology (CAST), “G. d’ Annunzio” University of Chieti-Pescara, Via L. Polacchi, 11, 66100 Chieti, Italy; 5 Unit of Medical Genetics, Department of Biomedical Sciences—BIOMORF, University of Messina, Via Consolare Valeria, 98125 Messina, Italy; alberti.saverio@gmail.com; 6 Sbarro Institute for Cancer Research and Molecular Medicine, Center for Biotechnology, Department of Biology, College of Science and Technology, Temple University, Philadelphia, PA 19122, USA

**Keywords:** pancreas, clear-cell primary adenocarcinoma, mitochondria, extracellular vesicles, transmission electron microscopy

## Abstract

Pancreatic cancer, most frequently as ductal adenocarcinoma (PDAC), is the third leading cause of cancer death. Clear-cell primary adenocarcinoma of the pancreas (CCCP) is a rare, aggressive, still poorly characterized subtype of PDAC. We report here a case of a 65-year-old male presenting with pancreatic neoplasia. A histochemical examination of the tumor showed large cells with clear and abundant intracytoplasmic vacuoles. The clear-cell foamy appearance was not related to the hyperproduction of mucins. Ultrastructural characterization with transmission electron microscopy revealed the massive presence of mitochondria in the clear-cell cytoplasm. The mitochondria showed disordered cristae and various degrees of loss of structural integrity. Immunohistochemistry staining for NADH dehydrogenase [ubiquinone] 1 alpha subcomplex, 4-like 2 (NDUFA4L2) proved specifically negative for the clear-cell tumor. Our ultrastructural and molecular data indicate that the clear-cell nature in CCCP is linked to the accumulation of disrupted mitochondria. We propose that this may impact on the origin and progression of this PDAC subtype.

## 1. Introduction

Pancreatic cancer (PC) is rapidly becoming the third leading cause of cancer death [1], thus representing an urgent, unmet medical need.

PC aggressiveness determinants are still poorly understood. Most patients are diagnosed at an advanced stage, only 10–15% of cases being suitable for curative surgical resection. Late diagnosis, large size, and early metastasis, together with chemoresistance and aggressive biology, have contributed to PC’s poor prognosis, with an overall survival of less than 5% [2].

Cellular and matrix epithelial–mesenchymal transition (EMT) determinants [3] have been identified as risk factors. Among them is the desmoplastic reaction, a fibrotic response that surrounds cancer cells and that can act as a barrier for chemotherapeutic agents [4]. Stromal cells such as cancer-associated fibroblasts, tumor-associated macrophages, and myeloid-derived suppressor cells contribute to generating a highly immunosuppressive tumor microenvironment (TME) [5].

The KRAS mutation is the most common genetic driver in pancreatic ductal adenocarcinoma (PDAC). The mutation landscape observed in PDAC cells includes loss-of-function mutations in tumor suppressor genes, such as TP53, CDNK2A, and DPC4/SMAD4. Additional mutations are detected in the AT-rich interaction domain 1A (ARID1A), breast cancer gene (BRCA) 1, and BRCA2 [6]. Mutations in BRCA genes can account for familial PC [7].

PDAC accounts for about 90% of all PC cases [8]. Based on histopathological features, PDACs have been subcategorized into seven variants: adenosquamous carcinoma, colloid carcinoma (mucinous non-cystic carcinoma), hepatoid carcinoma, medullary carcinoma, signet-ring-cell carcinoma, undifferentiated carcinoma, and undifferentiated carcinoma with osteoclast-like giant cells [6,9]. Clear-cell carcinoma of the pancreas (CCCP) is a rare morphological pattern of ductal adenocarcinoma that is not yet fully defined. It was first observed by Cubilla et al. in 1980 [10], and only a few cases have been reported since [11]. CCCP presents with tumor cells that have clear cytoplasm and hyperchromatic nuclei. Histochemical characterization using the PAS diastase reaction and mucicarmine staining revealed heterogeneous positivity for mucins [12,13,14]. Immunohistochemistry (IHC) has shown that the clear cells are of ductal origin due to their positivity for markers like cytokeratin (CK) 7, 8, 18, and 19.

Little information is available on the cellular biology of CCCP cells. Furthermore, no study has yet been conducted to explore the ultrastructure features of these neoplastic clear cells. In the present study, we explored CCCP cell ultrastructure and metabolic pathways, to gain insight into CCCP differentiation determinants.

## 2. Patient Report

In 2019, a 65-year-old man was admitted to the “P. Pederzoli” Hospital in Peschiera del Garda (VR, Italy) for the onset of type 2 diabetes mellitus. Serum biochemistry showed elevated tumor markers, i.e., CEA 2.9 ng/mL (normal 0–7.0 ng/mL) and CA-19.9 15.2 U/mL (normal: 0–37.0 U/mL). Genetic testing did not detect mutated BRCA1 or BRCA2 variants. Abdominal NMR showed a pancreatic mass of 25 × 19 mm at the body–tail boundary. Distal pancreatectomy and splenectomy were performed after 24 h. Surgical pathology analysis of a 25 mm-diameter mass revealed a moderately differentiated PDAC with the presence of clear cells. A tissue sample collected from the neoformation immediately after surgery was processed for transmission electron microscopy (TEM) analysis. No post-operative adjuvant chemotherapy was administered. The patient was discharged from the hospital at day 7 after surgery and was pronounced dead in 2021.

## 3. Results

### 3.1. Histopathology Findings

**Figure 1:**
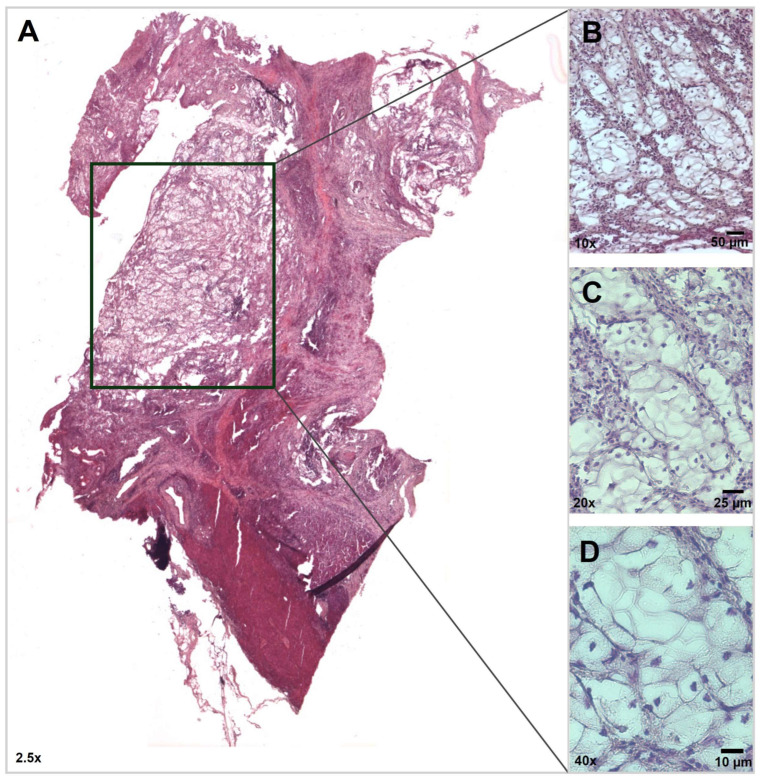
Histological characterization of the PDAC tissue sample (H&E staining). (**A**) Whole-slide analysis identifies a prominent area of clear-cell aspect (black box). (**B**–**D**) At higher magnifications, the tumor shows clear cells with a predominantly solid growth pattern. Scale bars are shown.

**Figure 2:**
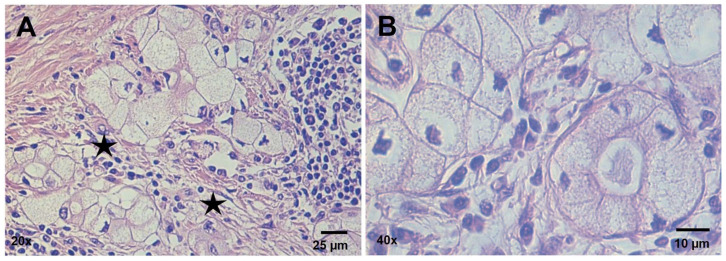
CCCP architecture (H&E staining). (**A**) Nests of clear cells immersed in a desmoplastic stroma (indicated by the black stars) near residual pancreatic acinar epithelial cells. (**B**) Higher magnification of the clear cells forming nest-like tumor structures. Scale bars are shown.

An overview of the histochemical characterization of the patient’s tissue sample is shown in Figure 1, with a special focus on the tumor areas (Figure 1B–D). Upon examination of the Hematoxylin and Eosin (H&E) staining of the formalin-fixed, paraffin-embedded (FFPE) pancreatic tumor tissue, neoplastic cells exhibited a prominent, clear-cell feature arranged to form nest-like structures immersed in a desmoplastic stroma (Figure 2A,B). According to the diagnostic criteria of Kim et al. [13], these findings were interpreted as a transitional trend of cancer cells towards the loss of differentiation.

The tumor cells were large, round to oval, and appeared packed with abundant intracytoplasmic vacuoles that provided a characteristic foamy appearance. Nuclei were centrally or peripherally placed and were often hyperchromatic or pleomorphic with irregular borders.

**Figure 3:**
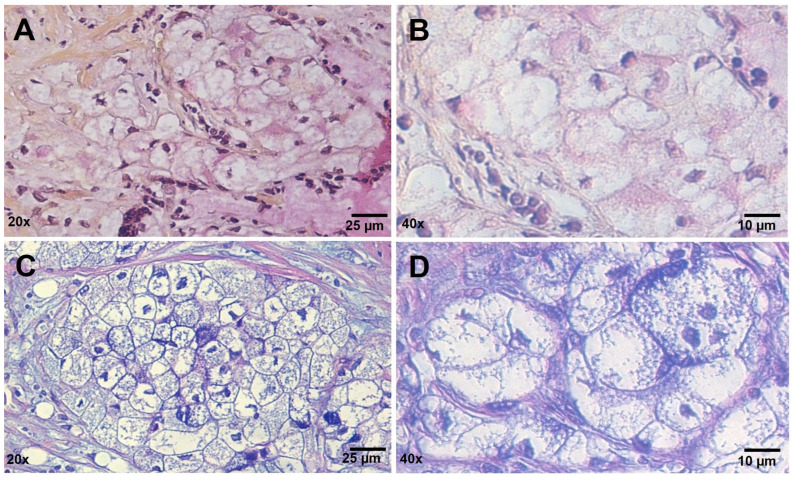
Mucin histochemical staining. (**A**,**B**) Mucicarmine staining; (**C**,**D**) Alcian–PAS reaction. Clear cells stained weakly, showing a characteristic thin positive texture within the clear cytoplasm. Scale bars are shown.

To investigate whether the clear-cell foamy appearance was due to an excess of mucins, mucicarmine and Alcian–PAS stainings were performed (Figure 3). Upon staining, the clear tumor cells showed heterogeneity, with some completely negative and others weakly stained, indicating the scant presence of acid mucin, if any. However, weakly stained cells showed a peculiar texture within the cytoplasm, also seen in previously described case reports but never thoroughly investigated [13].

Thus, to gain insight into the ultrastructure underlying this abundant clear cytoplasm that was not due to the accumulation of mucin vacuoles as in foamy gland adenocarcinomas, we went on to perform TEM and metabolic analysis of CCCP cells.

### 3.2. Ultrastructure of CCCP Cells

**Figure 4:**
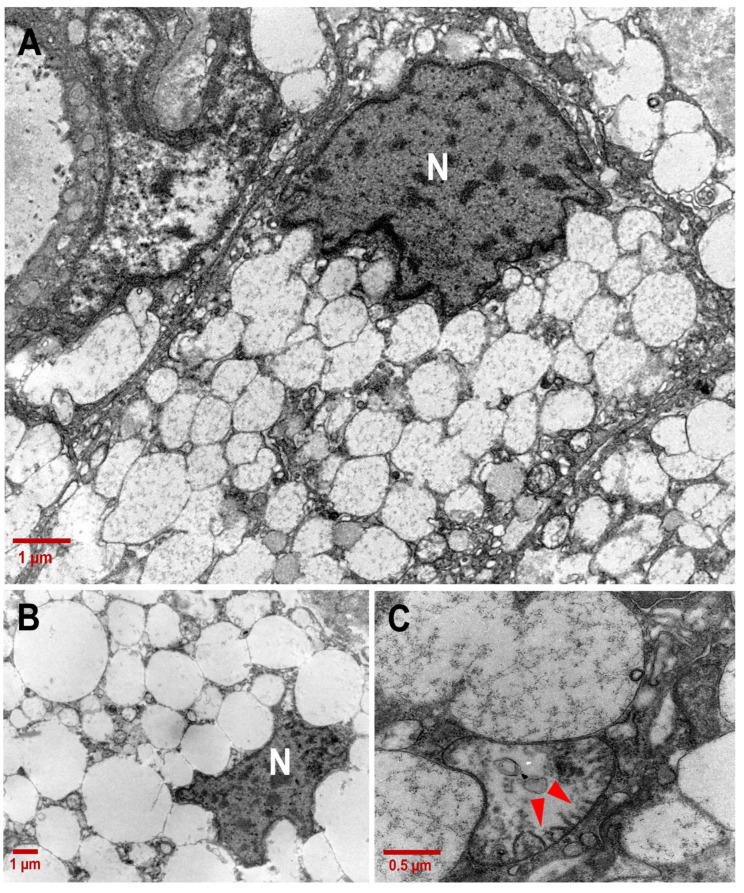
Ultrastructure of CCCP cell vacuoles. (**A**) Clear-cell vacuoles appeared partially electron-lucent under transmission electron microscopy (4400×; scale bar: 1 µm;). (**B**) Optically empty vacuoles imprinting the nuclear membrane. (**C**) Structures with the morphology of broken mitochondrial cristae (red arrow heads) were detectable in clear cells (20,000×; scale bar: 0.5 µm). N: nucleus.

The TEM analysis allowed us to to gather insight into the CCCP cytoplasmic organelle and vesicle distribution and content. While several intracytoplasmic vacuoles appeared devoid of detectable content, several others were characterized by poorly defined tubular structures that resembled mitochondrial cristae (Figure 4). The electron-lucent matrix combined with the irregular conformation of the cristae found inside these vacuoles indicated that they were mitochondria undergoing disruption. Consistent with this, cristae within the vacuoles appeared fragmented, suggesting cristolysis, as frequently observed during mitochondria swelling.

**Figure 5:**
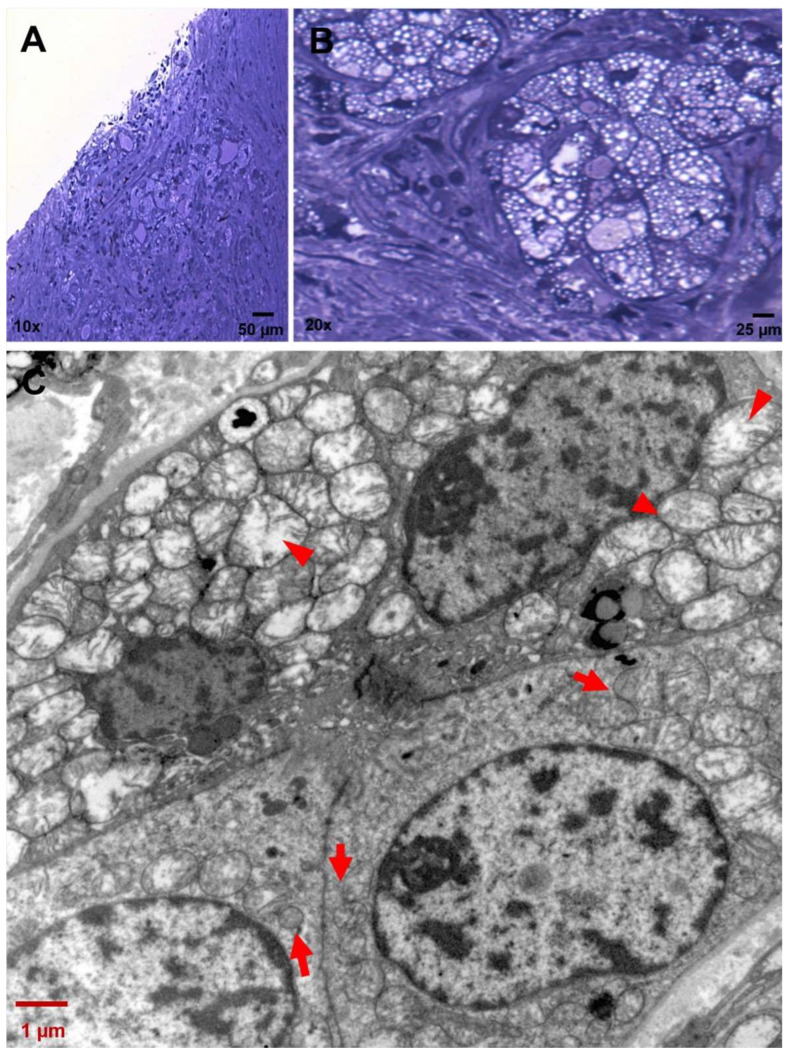
Sequential stages of mitochondrial degeneration in CCCP cells. (**A**,**B**) Toluidine-blue-stained semithin sections of a CCCP area. Magnification: 10×; 40×. (**C**) Sequence of mitochondrial involution stages within an acinus: from functional mitochondria with well-preserved cristae (red arrows) to swollen mitochondria with a progressive loss of integrity of cristae (red arrow heads) (4400×; scale bar: 1 µm).

The sequential steps of mitochondria degeneration were observed side by side in CCCP cells, suggesting sequential processes of progressive degeneration. In Figure 5B, four cells are shown with an acinar aspect and containing mitochondria at different morphological stages, from healthy mitochondria with well-preserved cristae to unhealthy mitochondria with completely disrupted cristae.

**Figure 6:**
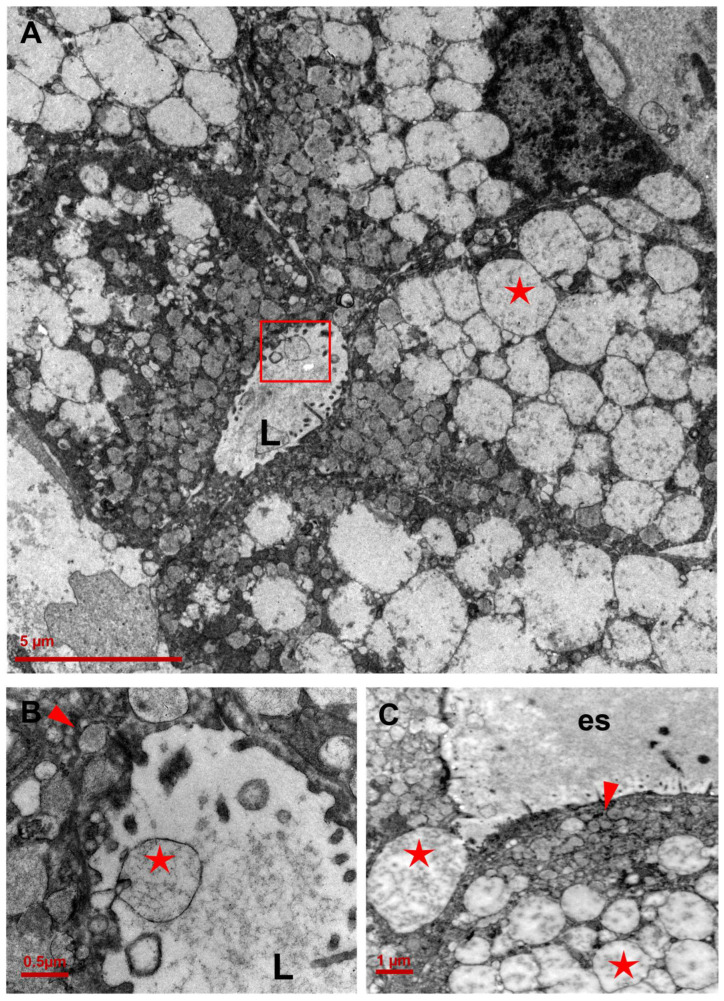
Vacuole distribution in CCCP nests. (**A**) Clear vacuoles detected both within the cells (red star) and the lumen of a neoplastic acinus (red box) (3000×; scale bar: 5 µm;). (**B**) Details of an external clear vacuole (red star). The red arrow head shows a cytoplasmic vacuole containing mucin (20,000×; scale bar: 0.5 µm). (**C**) In the apical portion of a clear cell, small electron-dense subplasmalemmal vacuoles containing mucin (red arrow head) and electron-lucent vacuoles derived by mitochondria (red stars) are easily distinguishable (4400×; scale bar: 1 µm;). L: lumen; es: extracellular space.

The diverse dimension, morphology, and electron density between subplasmalemmal vacuoles containing mucin and dysfunctional mitochondria is displayed in Figure 6. Unexpectedly, clear vacuoles were also found in the lumen of transformed acini (Figure 6B).

**Figure 7:**
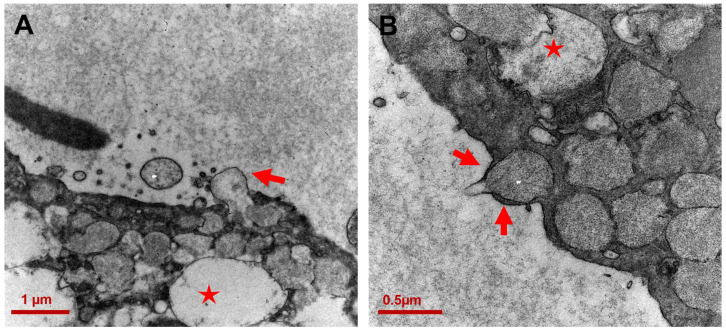
Transmembrane progression of vacuoles. (**A**) An electron-lucent vacuole appears emerging from the plasma membrane (red arrow) (12,000×; scale bar: 1 μm); (**B**) Protrusions resembling “arms” (red arrows) rise from the membrane to accommodate a mucin-vacuole (20,000×; scale bar 0.5 µm). Disrupted mitochondria are indicated by red stars.

Consistent with this finding, TEM captured membrane events (an example is shown in Figure 7A) where the cell seems to exocytose the cytoplasmic electron-lucent vacuole across the membrane into the extracellular space. Mucin-containing electron-dense vacuoles were also seen crossing the cell membrane (Figure 7B).

### 3.3. Immunohistochemistry for NDUFA4L2

**Figure 8:**
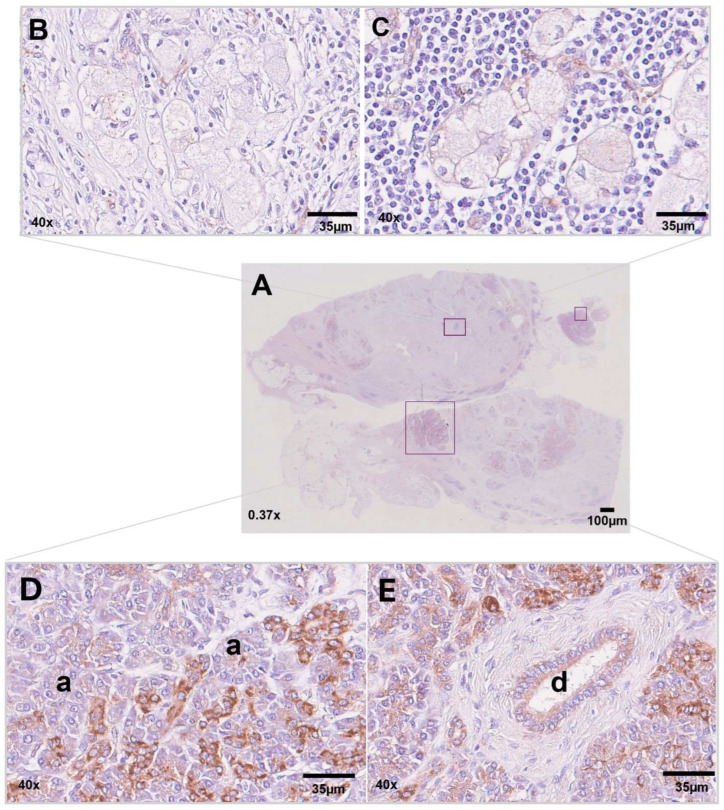
IHC analysis of NDUFA4L2 expression in CCCP cells. (**A**) Whole-slide scan. (**B**,**C**) CCCP showed virtually no NDUFA4L2 expression, with only a weak labeling detected in a few neoplastic cells. (**D**,**E**) Strong and diffuse NDUFA4L2 labeling was seen in the non-tumoral areas of the pancreatic tissue. a: acinar epithelial cells and centro-acinar cells; d: excretory duct. Scale bars are shown.

Based on our ultrastructural evaluation, intracytoplasmic vacuoles were indicated to correspond to unfunctional mitochondria with broken cristae. NADH dehydrogenase [ubiquinone] 1 alpha subcomplex, 4-like 2 (NDUFA4L2), is a subunit of Complex I of the mitochondrial respiratory chain, which is highly expressed in clear-cell renal carcinoma and involved in the transfer of electrons from NADH to ubiquinone and in the regulation of autophagic turnover of damaged mitochondria [15]. The IHC investigation of NDUFA4L2 expression in the CCCP tissue sample (Figure 8A) showed a weak labeling signal shaping the intracellular texture in a few neoplastic cells (Figure 8B,C), whereas most clear cells did not express NDUFA4L2. In contrast, NDUFA4L2 labeling was detected in non-neoplastic acinar and ductal epithelial cells (Figure 8D,E).

## 4. Discussion

Although data on incidence and follow-up are currently limited, previous reports have described CCCP as an aggressive histological variant of PDAC, associated with poor patient outcomes [11]. In this report, we applied ultrastructural TEM analysis to a CCCP surgical specimen from a 65-year-old patient. Our results show for the first time that the optical “clear cells” or foamy-cell variants of PDAC display ultrastructural hallmarks of altered mitochondria. Indeed, we discovered that the clear vacuoles within tumor cells correspond to swollen electron-lucent mitochondria, in the process of losing structure integrity and containing cristae remnants. These observations are unprecedented. It is known that several factors can lead to mitochondrial swelling, including oxidative and/or energetic stress such as free radical production, lipidic peroxidation, and hypoxia [16,17]. Mitochondrial swelling has been linked to the oxidation of the complexes of the oxidative chain and is correlated, together with mitochondrial crowding, with the accumulation of misfolded proteins inside mitochondria and the endoplasmic reticulum, causing the oxidation of mitochondrial proteases and chaperones [18].

Mitochondrial swelling and cytoplasmic vacuolization are also important signs of paraptosis, a non-apoptotic form of regulated cell death (RCD) first described by Sperandio et al. [19]. There is evidence that paraptosis is triggered when neoplastic cells are treated with specific compounds [20], but the real role of this alternative cell death in cancer has yet to be defined. The ultrastructural similarities found in our case suggest a link with paraptosis. This is not so unusual for cancer cells, considering that other forms of RCD, such as necroptosis, are emerging as strategies for tumor cell survival and growth, especially in PDAC [4].

Electron-lucent vacuoles were present inside the cells and in the lumen of neoplastic acini (Figure 6). The frequency of transitions across the plasma membrane suggests vacuole exchange between cells (Figure 6 and Figure 7). Under physiological conditions, mitochondrial transfer across cells is a common mechanism triggered by cells to maintain tissue homeostasis. It generally refers to the effective horizontal transport of functioning mtDNA copies or of entire mitochondria from donor cells, to restore the normal activity in the recipient hosts [21].

Both healthy and damaged mitochondria can be transferred among cells [22]. For example, within the bone marrow stem cell niche, the low oxygen environment induces mesenchymal stem cells (MSCs) to package partially depolarized mitochondria into larger vesicles (>100 nm), which are then accepted by surrounding macrophages [23]. This phenomenon is known as “trans-mitophagy” and allows the cell to “commission” the degradation of unfunctional organelles to recipient cells, after releasing them in the extracellular space. It is worth noting that the functionality of the mitochondria is irrelevant for their transfer, while intact mtDNA is indispensable for mitochondria to be transferred to another cell to recover the respiratory capacity of mitochondria-deficient cancer cells [24].

There are different modalities to “shuttle” mitochondria. Neighboring cells can connect each other by forming ultrafine cytoplasmic bridges called tunneling nanotubes (TNTs). Alternatively, they can establish a distance connection through the release of extracellular vesicles (EVs) containing the mitochondrial cargo, as was observed in our case. EVs are critical mediators of organized communities of cells, involved in several pathways such as differentiation, migration and immunoregulation [25]. In this case, we propose that CCCP could dispose of unfunctional mitochondria through a mechanism of trans-mitophagy, becoming a potential source of extracellular vesicles. The reason for this release is unknown, but given the main role of TME in PDAC, it could be an additional means of communication between the tumor and surrounding cells.

NDUFA4L2 mediates the function of oxidative phosphorylation, so it is critical for mitochondrial activity [26]. Loss of NDUFA4L2 through gene silencing leads to the inhibition of the autophagic machine and increased mitochondrial mass [15]. The hypothesis that clear cells could contain unfunctional mitochondria undergoing mitophagy is supported by the general absence of NDUFA4L2 in CCCP cells, except for sparsely positive groups. The opposite situation is seen within non-tumoral pancreatic structures that are significantly labeled by the anti-NDUFA4L2 antibody and might explain the different functional states of the mitochondria between normal and neoplastic cells.

Mitochondrial reprogramming is critical for tumorigenesis [27]. Hence, our findings suggest a link between mitochondrial disfunction and CCCP tumor progression.

## 5. Materials and Methods

### 5.1. Light Microscopy

Human samples of primary pancreatic ductal adenocarcinoma were collected immediately after surgical excision and fixed with 4% formaldehyde in 1× phosphate-buffered saline (PBS) for 24 h. After dehydration in graded alcohols (from 70% to 100% ethanol), samples were embedded in paraffin, and cut on a microtome (Leica, RM 2265, Nussloch, Germany). Sections were stained with Mayer’s hemallum (Bio-Optica, Milan, Italy) and 1% Eosin Y aqueous solution (Bio-Optica), Alcian Blue pH 2.5–PAS. (Bio-Optica), or Mucicarmine Mayer (Bio-Optica), according to the manufacturer’s instructions. MetaMorph 6.1 Software System (Universal imaging Corp, Molecular Device Corp, San Jose, CA, USA) with 2D reconstruction was employed to acquire digital images.

### 5.2. Immunohistochemistry (IHC)

The expression of NDUFA4L2 was evaluated with IHC on formalin-fixed, paraffin-embedded tissue (FFPE) sections. Tissue sections were incubated with a 1:80 dilution of anti-NDUFA4L2 rabbit polyclonal antibody (Proteintech, Chicago, IL, USA) for 90 min, followed by incubation with HRP-conjugated goat anti-rabbit secondary antibody (Thermo Fisher Scientific, Waltham, MA, USA) according to the manufacturer’s instructions. A validated skin tissue microarray was used as a staining control. Whole-slide images were acquired with NanoZoomerXR, Hamamatsu.

### 5.3. Transmission Electron Microscopy (TEM)

Following the protocol of Basile at al. [28], pancreatic ductal adenocarcinoma samples from surgical excision were fixed with 2.5% glutaraldehyde (Electron Microscopy Sciences, Hatfield, PA, USA) in 0.1 M cacodylate buffer (Electron Microscopy Sciences) (pH 7.2–7.4) for 2 h at 4 °C and post-fixed in 1% osmium tetroxide (Electron Microscopy Sciences) for 1 h at 4 °C. After a washing cycle in cacodylate buffer, samples were dehydrated in graded alcohols (from 50% to 100% ethanol) and embedded in Spurr resin (Electron Microscopy Sciences). Ultrathin sections (70 nm) were obtained by using a Reichert ultramicrotome (Reichert, Inc, Teramo, Italy) mounted on 200-mesh copper grids (Electron Microscopy Sciences) and counterstained with UranyLess and lead citrate staining solutions (Electron Microscopy Sciences) for 10–15 min/each. Samples were observed under a ZEISS EM109 electron microscope equipped with a Gatan 830Z00W44 camera (Gatan GmbH, Ingol-stadterstr. 12 D-80807 München, Germany). Digital Micrograph software version 2.02.800.0 was used for acquiring and processing digital image data [28].

## Data Availability

Data are contained within the article.
